# Endoscopic management of upper tract urothelial carcinoma with laser ablation including Magneto pulse modulation

**DOI:** 10.1002/bco2.70263

**Published:** 2026-08-27

**Authors:** Davide Perri, Luis Rico, Giorgio Bozzini, Pablo Contreras

**Affiliations:** ^1^ Department of Urology Azienda Socio Sanitaria Territoriale Lariana, Sant'Anna Hospital Como Italy; ^2^ Department of Urology Hospital Alemán Buenos Aires Argentina

**Keywords:** endoscopic ablation, Holmium, laser, Magneto, TFL, UTUC

## Abstract

**Objective:**

This study aimed to assess the efficacy and safety of different laser technologies, including the new Holmium:YAG (Ho:YAG) Magneto pulse modulation, for the endoscopic ablation of upper tract urothelial carcinoma (UTUC).

**Materials and methods:**

Sixty‐eight patients with UTUC from two centres were treated between February 2022 and June 2025. Three different lasers were used based on the available technology in each centre at the time of treatment: Ho:YAG with Vapor Tunnel pulse modulation (VT, Group A), thulium fibre laser (TFL, Group B) and Ho:YAG with Magneto pulse modulation (Mag, Group C). Intraoperative parameters, postoperative complications and recurrence rates were assessed.

**Results:**

Mean tumour size was 13.7 mm (range 5–30 mm). The overall mean operative time was 44.3 min. No intraoperative complications occurred. The overall rate of post‐procedural complications was 19.1%. The most frequent complication was haematuria, affecting 10.3% of patients. The recurrence rate was 17.6% (12 out of 68 patients) with a mean time to recurrence of 3.3 months. At the last follow‐up, 80.9% of patients were alive and free of disease, whereas 11.8% were alive with disease. Mortality due to urothelial carcinoma occurred in 1.5% of patients, whereas 5.8% of patients died from other causes.

**Conclusions:**

Endoscopic laser UTUC ablation is feasible and safe using both Ho:YAG and TFL, including the new Magneto pulse modulation technology. The low complication and recurrence rates support the use of this treatment in highly selected patients.

## 1 INTRODUCTION

Upper tract urothelial carcinoma (UTUC) is a rare tumour, accounting for 5%–10% of all urothelial cancers, with an annual incidence of 1–2 cases per 100 000 people.^1^, ^2^ Radical nephroureterectomy (RNU) is historically the gold standard treatment for UTUC, even in the case of localized disease.^1^ However, RNU is associated with significant loss of renal function, with a potential 20%–25% decrease in estimated glomerular filtration rate (eGFR) and a subsequent risk of developing chronic kidney disease, which can negatively impact overall survival.^3^, ^4^ Consequently, kidney‐sparing treatments like segmental ureterectomy or endoscopic ablation gained popularity in order to reduce the morbidity associated with RNU and preserve renal function without compromising oncological outcomes.^5^, ^6^

Guidelines from the European Association of Urology (EAU) recommend conservative management as a treatment option for patients with low‐risk tumours. These are typically defined as unifocal disease, less than 2 cm in size, low‐grade cytology, low‐grade ureteroscopic biopsy and absence of invasive features on computed tomography (CT) scan.^1^ Conservative management should also be considered in imperative cases for patients unsuitable for RNU, such as those with a solitary kidney, bilateral tumours, severely compromised renal insufficiency or multiple comorbidities.^7^

Endourology has witnessed many technological advances in the last years. Miniaturization of instruments, high‐quality digital flexible ureteroscopes and lasers with different physical properties have improved the quality of the endoscopic procedures, including the management of UTUC. Patients need to be carefully counselled given the need for stricter surveillance, the risk of disease downgrading and/or downstaging and the risk of disease recurrence or progression compared to RNU.^8^ Additionally, repeated diagnostic and operative ureteroscopic procedures must be taken into account for these patients.^9^

Holmium:YAG (Ho:YAG) laser was the first to be used for endoscopic UTUC ablation. Vapor Tunnel pulse modulation technology is based on a specific pulse modulation of the Ho:YAG laser, producing a single long pulse with minimal peak power. The initial part of the pulse creates a vapour channel, allowing the remaining energy to pass through it, resulting in finer stone ablation and reduced retropulsion.^10^, ^11^ Thulium fibre laser (TFL) has been praised for its shorter wavelength, lower penetration depth, lower peak and longer duration of its pulses, as well as the possibility to switch from pulsed to continuous wave output. Initial studies have shown promising results in terms of safety and efficacy, with low recurrence and complication rates.^6^ The latest arrival in the endourological panorama is represented by a new pulse modulation technology called Magneto, which has been added to the already existing high‐power Ho:YAG Cyber Ho laser generator (Quanta System, Samarate, Italy). Magneto technology enables modification of the shape of laser pulses by increasing their duration up to 2000 μs and decreasing peak power down to 500 W. Initial experiences on stone dusting^12^, ^13^, ^14^ and prostate enucleation, including comparative studies,^15^ have already been published, showing promising results. However, data on UTUC ablation are absent.

Although endoscopic UTUC ablation is by now an already established alternative for low‐risk patients, most reports from the literature are limited by very small single‐centre cohorts, often with short‐term follow‐up. In this study, we present a large multicentre cohort of patients to assess the efficacy and safety of UTUC ablation with different lasers, including the first data on the use of Magneto technology.

## 2 MATERIALS AND METHODS

Patients included had a diagnosis of low‐risk UTUC according to EAU guidelines: unifocal disease, less than 2 cm in size, low‐grade cytology, low‐grade ureteroscopic biopsy and absence of invasive features on CT scan.^1^ A high‐risk group of patients with solitary kidney who had a high‐grade biopsy was also included. Patients with coagulation issues or those on anticoagulant therapy that could not be temporarily stopped were excluded. Patients were retrospectively reviewed from two centres (Sant'Anna Hospital, Como, Italy; Hospital Alemán, Buenos Aires, Argentina), and the treatment period of time was between February 2022 and June 2025. All patients underwent a complete preoperative workup, including thoraco‐abdominal CT scan/MRI, urine cytology and cystoscopy to rule out bladder lesions. In case of bladder tumour, the patient was not excluded, and a transurethral resection was performed at the end of the procedure.

A 7‐Fr semirigid ureteroscope (Karl Storz, Tuttlingen, Germany) was used to get access to the ureteral lumen. The ureter was checked in every case, also when the preoperative imaging had shown the lesion inside the renal cavity. The same instrument was used to perform the laser ablation when the lesion was located in the ureter. For pelvic, calyceal or proximal ureteral lesions, a digital flexible ureteroscope (FLEX‐XC1, Karl Storz) was used after placing a ureteral access sheath (Bi‐Flex Evo, Rocamed, Monaco, France). The access sheath was used in all cases of intrarenal lesions in order to facilitate access and minimize the risk of tumour cell dissemination. Irrigation was gravity‐fed, with saline bags positioned nearly 50 cm above the patient. Biopsies were performed before the laser ablation using forceps for flat lesions (Biopsy Forceps, Karl Storz or Pirahna Forceps, Boston Scientific, Marlborough, Massachusetts, USA) and a basket for exophytic lesions (ZeroTip; Boston Scientific, Marlborough, Massachusetts, USA). At the end of the procedure, a ureteral stent was always placed. All patients underwent cystoscopy, urine cytology and abdominal CT scan 3 and 6 months after surgery. Afterwards, cystoscopy was performed every 6 months. Abdominal imaging was obtained by alternating ultrasound and CT scan every 6 months. Intraoperative parameters, perioperative complications and pathological and oncological outcomes were assessed. Recurrence was considered a new diagnosis of UTUC.

Three different lasers were used to perform the tumour ablation based on the available technology in each centre at the time of treatment: Ho:YAG LithoEVO generator with Vapor Tunnel pulse modulation (VT, Group A), TFL Fibre Dust generator set in pulsed‐wave mode (TFL, Group B) and Ho:YAG Cyber Ho generator with Magneto pulse modulation (Mag, Group C). Laser settings were not always the same in both centres for every case. However, a strict range of parameters was used. Specifically, energy ranged from 0.6 to 1 J, frequency was fixed at 10 Hz and total power ranged from 6 to 10 W. A 200‐ or 272‐μm laser fibre was used in all cases.

Mean and standard deviation (SD) versus numbers and proportions were used to describe continuous and categorical variables, respectively. To assess for significant differences in operative time among the three lasers, a one‐way analysis of variance (ANOVA) was employed. The assumption of homogeneity of variances was evaluated using Levene's test prior to the analysis. The comparison of postoperative complication rates across the three laser groups was performed using a Fisher's exact test. A two‐sided *p* < 0.05 was considered statistically significant. All statistical analyses were performed using GraphPad Prism (version 9.5.1).

## 3 RESULTS

### 3.1 Study population

Sixty‐eight consecutive patients were included and retrospectively reviewed from both centres. The mean age was 76.9 years. Six patients (8.8%) had a solitary kidney and were given an imperative indication for endoscopic laser ablation. Mean preoperative creatinine (Cr) was 1.5 mg/dL, with a mean eGFR of 65.9 mL/min. Overall, 36 patients (52.9%) were diagnosed with a urothelial carcinoma for the first time, 29 (42.6%) had a previous bladder cancer and 2 (2.9%) had a previous history of UTUC. In one case (1.5%), the patient had a previous history of bladder cancer and UTUC. The mean tumour size was 13.7 mm (range 5–30 mm). The majority of patients (56, 82.4%) had a single lesion, whereas 11 patients (16.2%) had two lesions and 1 patient (1.5%) had three lesions. The most frequent tumour location was the renal pelvis (31 patients, 45.6%), followed by the renal calyx (21 patients, 30.9%) and the ureter (15 patients, 22.1%). One patient (1.5%) had two simultaneous lesions located in the renal pelvis and the ureter. Urine cytology examination resulted as negative in 30 cases (44.1%), positive for low‐grade tumour in 29 cases (52.6%) and positive for high‐grade tumour in 9 cases (13.2%). Preoperative cystoscopy revealed the presence of a bladder lesion in 14 patients (20.6%). Laser ablation was performed with Ho:YAG laser and VT pulse modulation in 20 cases (29.4%), with TFL in 24 cases (35.3%) and with Ho:YAG laser with Magneto pulse modulation in 24 cases (35.3%) (Table 1).

**TABLE 1:** Descriptive characteristics of 68 patients with a diagnosis of UTUC who underwent endoscopic laser ablation.

		UTUC laser ablation (*n* = 68)
Age, years	Mean (SD)	76.9 (8.4)
Gender	*n* (%)	
Male		52 (76.5)
Female		16 (23.5)
Solitary kidney	*n* (%)	6 (8.8)
Preoperative Cr	Mean (SD)	1.5 (0.7)
Preoperative eGFR	Mean (SD)	65.9 (15.3)
Clinical status	*n* (%)	
First diagnosis		36 (52.9)
Previous bladder cancer		29 (42.6)
Previous UTUC		2 (2.9)
Previous UTUC + bladder cancer		1 (1.5)
Tumour size, mm	Mean (SD)	13.7 (6.4)
	Range	5–30
Tumour location	*n* (%)	
Renal pelvis		31 (45.6)
Renal calyx		21 (30.9)
Ureter		15 (22.1)
Renal pelvis + ureter		1 (1.5)
Number of lesions	*n* (%)	
1 lesion		56 (82.4)
2 lesions		11 (16.2)
3 lesions		1 (1.5)
Urine cytology	*n* (%)	
Negative		30 (44.1)
Low grade		29 (52.6)
High grade		9 (13.2)
Cystoscopy	*n* (%)	
Negative		54 (79.4)
Positive		14 (20.6)
Laser ablation	*n* (%)	
Ho:YAG VT		20 (29.4)
TFL		24 (35.3)
Ho:YAG Mag		24 (35.3)

Abbreviations: Cr, creatinine; eGFR, estimated glomerular filtration rate; Ho:YAG, Holmium:YAG; Mag, Magneto; SD, standard deviation; TFL, thulium fibre laser; UTUC, upper tract urothelial carcinoma; VT, Vapor Tunnel.

### 3.2 Perioperative outcomes

The overall mean operative time was 44.3 min. Specifically, mean operative time was 43.4 min with Ho:YAG laser and VT pulse modulation, 43.5 min with TFL and 44.7 min with Ho:YAG laser and Magneto pulse modulation. A one‐way ANOVA test was performed, and the result, with an *F*‐statistic of 0.154 and a *p*‐value of 0.85, showed that there was no statistically significant difference among the groups. No intraoperative complications occurred. The overall rate of post‐procedural complications was 19.1%. The most frequent complication was haematuria, affecting 10.3% of patients. Haematuria was managed through increased oral hydration and prolonged catheterization whenever needed. No patients needed blood transfusions or embolization. The second most common complication (4.4%) was stent discomfort requiring the use of analgesics, followed by urinary tract infection (2.9%), which was solved through prolonged antibiotic therapy. All these complications were grade I according to the Clavien–Dindo classification. One patient (1.5%) developed a postoperative ureteral stricture, which was managed with an endoscopic balloon dilation. The differences in complication rates among the three groups were not statistically significant (Fisher's exact test, *p* = 0.35) (Table 2).

**TABLE 2:** Intraoperative and postoperative outcomes of 68 patients with a diagnosis of UTUC who underwent endoscopic laser ablation.

		UTUC laser ablation (*n* = 68)
Operative time, min	Mean (SD)	
Overall		44.3 (10.2)
VT		43.4 (10.5)
TFL		43.5 (10.6)
Mag		44.7 (10.1)
Haematuria	*n* (%)	
Overall		7 (10.3)
VT		1 (1.5)
TFL		‐
Mag		6 (8.8)
Stent discomfort	*n* (%)	
Overall		3 (4.4)
VT		1 (1.5)
TFL		2 (2.9)
Mag		‐
Urinary tract infection	*n* (%)	
Overall		2 (2.9)
VT		2 (2.9)
TFL		‐
Mag		‐
Ureteral stricture (TFL)	*n* (%)	1 (1.5)

Abbreviations: Mag, Magneto; SD, standard deviation; TFL, thulium fibre laser; UTUC, upper tract urothelial carcinoma; VT, Vapor Tunnel.

### 3.3 Oncological outcomes

**FIGURE 1:**
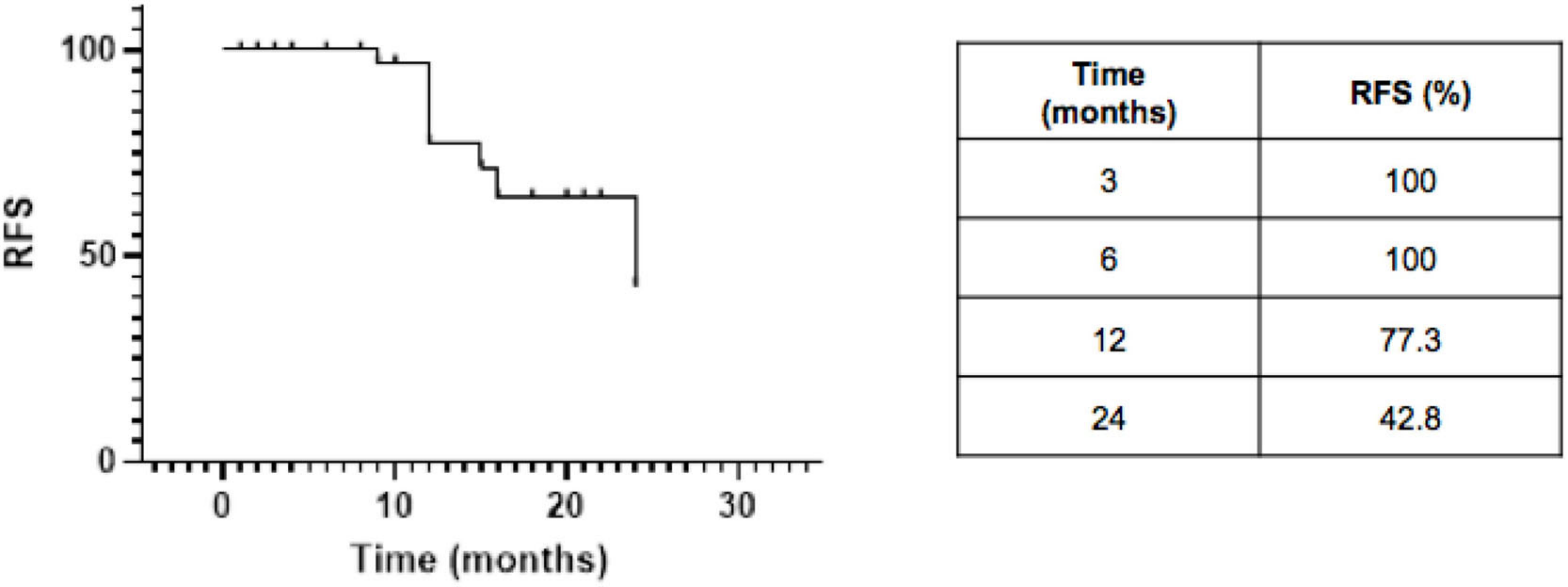
Kaplan–Meier curve showing recurrence‐free survival (RFS) of 68 patients with a diagnosis of upper tract urothelial carcinoma who underwent endoscopic laser ablation.

Histopathological evaluation confirmed a urothelial carcinoma in all cases. Tumour grade was low versus high in 55 (80.9%) versus 13 (19.1%) cases. The mean follow‐up period was 16.0 months (range 6–42). Overall, the UTUC recurrence rate was 17.6% (12 out of 68 patients) with a mean time to recurrence of 3.3 months. The site of recurrence was the same as the first tumour in the majority of cases (eight patients, 66.7%), whereas in four cases (33.3%), the recurrence was located elsewhere but in the same upper tract. Different types of treatment were planned for the recurrences. Specifically, one patient (8.3%) underwent distal ureterectomy, seven patients (58.3%) underwent endoscopic laser ablation and four patients (33.4%) were treated with RNU. Figure 1 shows the Kaplan–Meier curve for disease‐free survival (DFS). Recurrence‐free survival (RFS) rates were 100% at 3 and 6 months, 77.3% at 12 months and 42.8% at 24 months. At the last follow‐up, 80.9% of patients were alive and free of disease, whereas 11.8% were alive with disease. Mortality due to urothelial carcinoma occurred in 1.5% of patients, whereas 5.8% of patients died from other causes. At last follow‐up, mean Cr was 1.6 mg/dL and mean eGFR was 65.6 mL/min (Table 3).

**TABLE 3:** Oncological outcomes of 68 patients with a diagnosis of UTUC who underwent endoscopic laser ablation.

		UTUC laser ablation (*n* = 68)
Tumour grade	*n* (%)	
Low grade		55 (80.9)
High grade		13 (19.1)
Follow‐up	Mean (SD)	16.0 (11.9)
	Range	(6–42)
Recurrence	*n* (%)	12 (17.6)
Time to recurrence	Mean (SD)	3.3 (0.2)
Recurrence site	*n* (%)	
Same location		8 (66.7)
Other location		4 (33.3)
Recurrence treatment	*n* (%)	
Distal ureterectomy		1 (8.3)
Endoscopic ablation		7 (58.3)
RNU		4 (33.4)
Status at last follow‐up	*n* (%)	
Alive without disease		55 (80.9)
Alive with urothelial cancer		8 (11.8)
Dead for urothelial cancer		1 (1.5)
Dead for other causes		4 (5.8)
Cr at last follow‐up	Mean (SD)	1.6 (0.7)
eGFR at last follow‐up	Mean (SD)	65.6 (15.5)

Abbreviations: Cr, creatinine; eGFR, estimated glomerular filtration rate; RNU, radical nephroureterectomy; SD, standard deviation; UTUC, upper tract urothelial carcinoma.

## 4 DISCUSSION

Despite RNU being the standard treatment for UTUC, it may be overtreatment for small, low‐grade tumours. The EAU guidelines recommend kidney‐sparing management as a potential primary option for patients with low‐risk tumours and, if technically feasible, also for patients with high‐grade tumours and imperative indications such as solitary kidney, bilateral UTUC or pre‐existing severe chronic kidney disease.^1^ Semirigid or flexible ureteroscopy with laser ablation has emerged as a promising conservative solution. Technological improvements have provided high‐quality instruments in terms of vision, flexibility, diameter, range of motion and laser devices. Many lasers are nowadays available, with different physical features and tissue interactions. Further, some of these lasers allow modification of the interaction with tissues through specific pulse modulation technologies. Taking into account the available lasers, the possible modifications of the shape of pulses and the wide range of setting combinations, the same treatment can be performed in many different ways. However, there are still no guidelines on which laser technology is best for UTUC ablation. Compared to stone treatment, studies in the literature are few, often with small single‐centre cohorts with short follow‐up. The aim of our study was to describe the outcomes of a relatively large cohort of patients who underwent UTUC ablation in two centres, using different lasers but applying the same technique, to enhance the available evidence on this treatment. Moreover, our study includes the first multicentre data on Magneto technology for UTUC ablation.

Mean operative time was comparable between the three groups. Notably, most studies focus on oncological outcomes with a lack of data regarding intraoperative and postoperative parameters.^16^, ^17^ Operative time may be seen as a surrogate of laser reliability in treating the tumour. Indeed, lasers have a different interaction with soft tissues. Even the same laser interacts differently according to the pulse modulation setting applied, like Vapor Tunnel versus Magneto in our study. Consequently, bleeding and haemostatic effect can be different. From this point of view, our data suggest that the physical properties of the lasers used do not translate into meaningful differences in procedural efficacy and therefore in operative time.

Similarly, the overall rate of postoperative complications was comparable. The majority of the reported complications were low grade and managed conservatively. Although the Magneto group had a slightly higher number of complications, a Fisher's exact test revealed that the difference was not statistically significant. Specifically, six patients in the Magneto group and one in the VT group showed haematuria postoperatively. This could suggest better haemostatic control with TFL. However, no patients required transfusions and the reported haematuria had no clinical impact for any of the above‐mentioned cases. The rates and typology of complications were overall comparable to those described from other published cohorts.^17^, ^18^, ^19^ Once more, these data underline the comparable safety profile of all the lasers used.

The main concern with any conservative surgical procedure is the increased risk of recurrence compared to radical treatments. Our study showed an overall recurrence rate of 17.6% (12 out of 68 patients) with a mean follow‐up of 16 months. This rate is in line with existing literature. Specifically, our recurrence rate is comparable to that reported by Bozzini et al. for Tm:YAG laser ablation (19.2% after a mean follow‐up of 11.7 months)^17^ and by Musi et al. (19% with an estimated RFS of 44 months).^20^ Our findings are also consistent with the initial data on TFL from Proietti et al., who reported a recurrence rate of 17.7% at 12‐month follow‐up.^19^ On the contrary, Villa et al. reported a higher recurrence rate of 51.2% after Ho:YAG laser ablation.^9^ In 2018, Wen et al. performed a retrospective comparison study on 32 UTUC patients conservatively treated with TFL versus RNU. They reported that the loss of renal function was lower in the conservative group, but the tumour recurrence rate was higher (21.9% vs. 7.8%).^21^ Defidio et al. described their experience of UTUC patients treated using a combination of dual wavelengths of Tm:YAG and Ho:YAG lasers. At last follow‐up, 69.3% of patients were recurrence‐free, 21.8% had endoscopically treated recurrences and a further 8.9% had undergone RNU, concluding that the Tm–Ho:YAG duo laser was safe and oncologically non‐inferior to the alternative laser energy technology for conservative UTUC treatment.^22^

The tumour location may play a role in the recurrence rate. Shvero et al. observed a higher incidence of local recurrence in patients with intrarenal tumours, attributing it to the continuous circulation of irrigation fluid and the risk of tumour seeding.^5^ This reinforces the importance of using a ureteral access sheath to minimize the risk of tumour cell dissemination. Notably, in our study, eight recurrences occurred in the same location of the initial tumour, whereas in the remaining four cases, the recurrence was located elsewhere within the same upper tract. This aspect deserves consideration. Whereas a same‐site recurrence can be a consequence of the ineffectiveness of the first conservative procedure, a recurrence in another site might not. Therefore, the recurrence rate should be more carefully evaluated.

Despite its strengths, some limitations of the study need to be acknowledged. First, the relatively low number of patients and the limited follow‐up need to be considered. Despite most studies on this topic in the literature having fewer patients, larger cohorts with a longer follow‐up are needed. Second, more surgeons were involved. This could influence results, as UTUC ablation may be challenging. However, all the involved urologists had a high level of expertise in endourology. Third, other lasers and pulse modulation technologies are available and were not included in the present study. Once more, larger multicentre studies are needed. Fourth, second‐look ureteroscopy was not systematically performed in all cases. This might have influenced the recurrence rate. Lastly, we did not compare the oncological outcomes between the three groups. However, our purpose was to assess the feasibility of the lasers used in UTUC ablation in terms of efficacy and safety, including the new Magneto technology. Oncological outcomes should be compared with more and longer term data. Moreover, the relationship between laser, pulse modulation and settings used with oncological outcomes is still to be carefully defined.^23^

## AUTHOR CONTRIBUTIONS

**Davide Perri:** Manuscript writing/editing, Data analysis. **Luis Rico:** Data analysis. **Giorgio Bozzini:** Protocol/project development, Supervision. **Pablo Contreras:** Protocol/project development, Supervision.

## CONFLICT OF INTEREST STATEMENT

All authors have no direct or indirect commercial financial incentive associated with publishing the article. There is no source of extra‐institutional funding.

## Data Availability

The data that support the findings of this study are available from the corresponding author upon reasonable request.
